# Pleomorphic adenoma with squamous and appendageal metaplasia mimicking mucoepidermoid carcinoma on cytology

**DOI:** 10.4103/1742-6413.45496

**Published:** 2008-02-12

**Authors:** Meenakshi Batrani, Manju Kaushal, A. K. Sen, Rajbala Yadav, N. K. Chaturvedi

**Affiliations:** Department of Pathology, Dr. Ram Manohar Lohia Hospital, New Delhi, India

**Keywords:** Mucoepidermoid carcinoma, pleomorphic adenoma, squamous metaplasia

## Abstract

**Background:**

Histological diversity is the hallmark of pleomorphic adenoma, the most common salivary gland tumor. It may cause difficulty in cytological interpretation, due to limited and selective sampling.

**Case presentation:**

A 16-year-old female patient presented with right cheek swelling. Fine needle aspiration cytology showed squamous cells, basaloid cells, and foamy cells, along with extracellular keratin and foreign body giant cells. Characteristic metachromatic fibrillary chondromyxoid stroma, which is usually seen in pleomorphic adenoma, was not seen in the aspirate. A diagnosis of mucoepidermoid carcinoma was given on cytology. Subsequent resection revealed an encapsulated pleomorphic adenoma, with extensive squamous metaplasia and appendageal differentiation on histology.

**Conclusion:**

This case illustrates that pleomorphic adenoma with squamous metaplasia presents a potential for misinterpretation as mucoepidermoid carcinoma on cytology. We discuss the various pitfalls and the features that are helpful in distinguishing these two lesions.

## BACKGROUND

Pleomorphic adenomas are easily identified on cytology, because of their characteristic biphasic pattern comprising of epithelial/myoepithelial cells and fibromyxochondroid stroma.[1] Fine needle aspiration cytology (FNAC) is a highly accurate tool for the diagnosis of pleomorphic adenoma, with a reported reliability of 80-95%.[1,2] However, even this common salivary gland neoplasm can be diagnostically challenging and cause pitfalls in cytodiagnosis. The presence of squamous metaplasia, especially in the absence of chondromyxoid stroma, may be misinterpreted as mucoepidermoid carcinoma.

## CASE PRESENTATION

### Brief clinical details

The patient, a I6-year-old female, presented with complaint of right cheek swelling since three years. The swelling was painless and progressively increasing in size. On examination, a 1.5 × 1.5 cm swelling was seen on both the outer aspect and the corresponding inner mucosal aspect of the right cheek. The swelling was well defined, firm, and mobile, with normal overlying skin.

### Cytological findings

**Figure 1:**
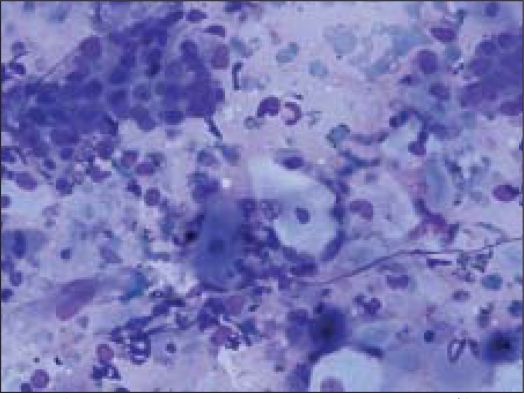
Cellular smear showing squamous and basaloid cells (Giemsa, ×100×)

**Figure 2:**
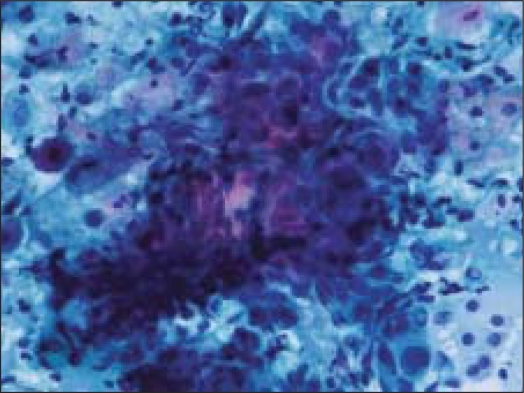
Cluster of basaloid cells with mild nuclear atypia (Papanicoloau stain, ×200×)

**Figure 3:**
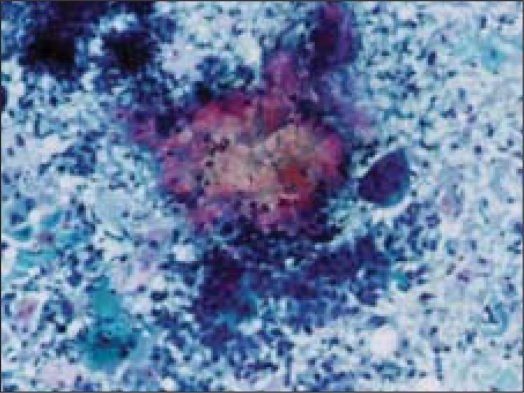
Whorls of extracellular keratin, with foreign body giant cells (Papanicoloau stain, × 100×)

**Figure 4:**
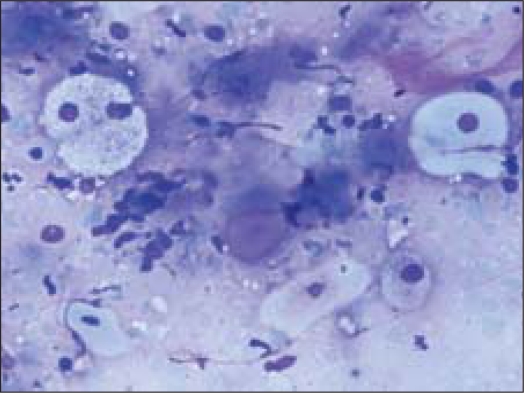
Vacuolated cells (Giemsa, ×200×)

Fine needle aspiration (FNA) was done using a 23 gauze needle attached to a 10 cc syringe. Both alcohol fixed and air dried smears, stained with Papanicolaou and Giemsa stains respectively, were examined. The smears showed a cellular aspirate with squamous and basaloid cells seen isolated and in clusters [Figure 1]. A few cells showed nuclear atypia [Figure 2]. Whorls of extracellular keratin, with foreign body giant cell reaction were seen [Figure 3]. Many vacuolated and foamy cells were also seen in a background of scant mucin and proteinaceous debris [Figure 4]. Metachromatic fibrillary chondromyxoid stroma, characteristic of pleomorphic adenoma, was not seen. A diagnosis of mucoepidermoid carcinoma was made, based on the cytological findings.

### Gross and histopathological findings

**Figure 5:**
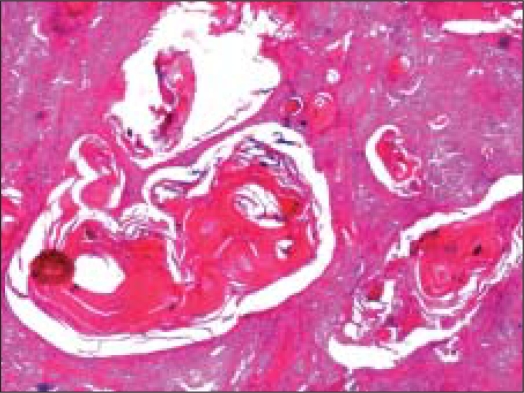
Sheets of squamous cells with multiple keratin filled cysts reminiscent of trichoepitheliomatous differentiation (Hematoxlin and eosin, ×40×)

**Figure 6:**
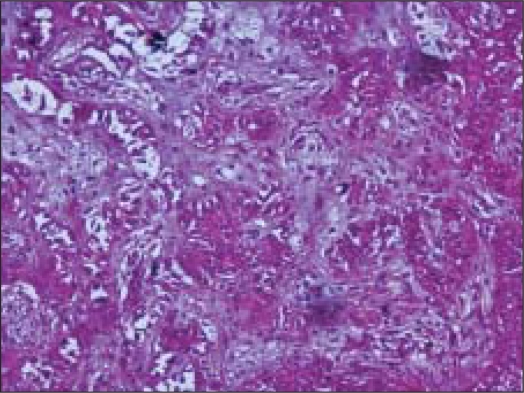
Areas of conventional pleomorphic adenoma (Hematoxlin and eosin, ×100×)

Surgical resection was done. Gross specimen comprised of an encapsulated soft tissue mass, measuring 1.5 cm in diameter. Cut surface was firm and grey white. No areas of hemorrhage, necrosis or cystic change were seen. Histology showed an encapsulated tumor mass with 75% of the tumor volume comprising of sheets of squamous cells, with multiple keratin filled cysts, reminiscent of trichoepitheliomatous differentiation [Figure 5]. The rest of the areas showed features of conventional pleomorphic adenoma [Figure 6]. A diagnosis of pleomorphic adenoma, with extensive squamous metaplasia and appendageal differentiation, was made.

## DISCUSSION

Histological diversity is the hallmark of pleomorphic adenoma.[3] Histological patterns vary considerably between different parts of the same tumor.[3] Not only does the proportion between epithelial and chondromyxoid stroma vary, but there are also metaplastic variations in the epithelial and stromal components.[4] Morphological diversity is often not a problem in surgical pathology, where the whole tumor is available for examination. However, this can lead to a misdiagnosis on cytology, due to limited and selective sampling.[3,4]

Focal squamous metaplasia is found in about 25% of pleomorphic adenomas. Rarely, florid squamous metaplasia is reported.[5] Adenexal differentiation in the form of extensive keratin filled cysts, reminiscent of trichoepitheliomatous differentiation, as in our case, is also reported in three other cases published in literature.[6–8]

Potential for misdiagnosis of pleomorphic adenoma as mucoepidermoid carcinoma on cytology include squamous and basaloid cells mimicking squamous and intermediate cells of mucoepidermoid carcinoma; presence of sebaceous/mucinous metaplastic cells, vacuolated histiocytic cells and mucoid material; and, absence of metachromatic fibrillar stromal material that characterizes most pleomorphic adenomas on aspiration cytology.[1,6,7]

In our case also, these features led to the cytological diagnosis of mucoepidermoid carcinoma. Similar cases are being reported in literature, where the cytological diagnosis of pleomorphic adenoma are given as mucoepidermoid carcinoma either as the only diagnosis or as a differential diagnosis [Table 1]. In a case report by Hamdan *et al.*, a misdiagnosis of mucoepidermoid carcinoma was made on frozen section.[9]

**Table 1:** Previous reported cases of pleomorphic adenoma with squamous metaplasia mimicking mucoepidermoid carcinoma

*Authors*	*Number of cases*	*Cytological diagnosis*
Orell *et al.*[4]	2	Mucoepidermoid carcinoma as differential
Vigeur *et al.*[2]	1	Low grade mucoepidermoid carcinoma
Lam K Y[5]	1	Differential of low grade mucoepidermoid
		Carcinoma and well differentiated squamous cell carcinoma
Kusum Verma[1]	5*	Mucoepidermoid carcinoma
Brachetel *et al.*[6]	1	Atypical neoplasm, cannot rule out mucoepidermoid carcinoma

* After review, three of the five cases were given as pleomorphic adenoma.

To avoid misinterpretation of pleomorphic adenoma with squamous metaplasia as mucoepidermoid carcinoma on cytology, a close scrutiny for fragments of chondromyxoid stroma, a characteristic feature for pleomorphic adenoma, is important.[4] In our case also, on reviewing the slides again after histological diagnosis, we could find occasional tiny fragment of stroma. Also, keratinization, especially of the extracellular type, is rare in mucoepidermoid carcinoma.[6] However, even if the features diagnostic of pleomorphic adenoma are identified, the differential diagnosis may still include a mucoepidermoid carcinoma arising in a preexisting pleomorphic adenoma. However, mucoepidermoid carcinoma ex pleomorphic adenoma is exceedingly rare and is usually a high grade malignancy.[6,7]

## CONCLUSION

Although FNAC is a highly accurate tool for the diagnosis of pleomorphic adenoma, the diagnosis of this common tumor can be challenging and cause pitfalls in cytodiagnosis. The presence of squamous, mucinous or sebaceous metaplasia, especially in the absence of chondromyxoid stroma, may be misinterpreted as mucoepidermoid carcinoma on cytology. Awareness of the cytological variations is important, so as to avoid diagnostic errors.

## COMPETING INTEREST STATEMENT BY ALL AUTHORS

No competing interest to declare by any of the authors.

## AUTHORSHIP STATEMENT BY ALL AUTHORS

According to International Committee of Medical Journal Editors (ICMJE http://www.icmje.org) “author” is generally considered to be someone who has made substantive intellectual contributions to a published study.

Authorship credit should be based on 1) substantial contributions to conception and design, acquisition of data, or analysis and interpretation of data; 2) drafting the article or revising it critically for important intellectual content; and 3) final approval of the version to be published. Authors should meet conditions 1, 2, and 3. Other contributors, who do not meet these criteria for authorship, are listed in an acknowledgments section.

All authors of this article declare that we qualify for authorship as defined by ICMJE http://www.icmje.org/#author.

Each author has participated sufficiently in the work and take public responsibility for appropriate portions of the content of this article.

MB conceived the study, performed the FNAC, performed literature search and drafted, proof read and scrutinized the manuscript.

MK participated in the diagnostic workup, helped in drafting the manuscript and proof reading.

AKS participated in the diagnostic workup.

NKC participated in the diagnostic workup, helped in drafting the manuscript and scrutinized the manuscript.

RBY helped in drafting the manuscript and scrutinized the manuscript.

All authors read and approved the final manuscript

Each author acknowledges that this final version was read and approved.
